# IGFBP2 is a potential biomarker in acute kidney injury (AKI) and resveratrol-loaded nanoparticles prevent AKI

**DOI:** 10.18632/oncotarget.25663

**Published:** 2018-11-27

**Authors:** Hai-Lun Li, Zhuan Yan, Zun-Ping Ke, Xiao-Feng Tian, Li-Li Zhong, Yong-Tao Lin, Yong Xu, Dong-Hui Zheng

**Affiliations:** ^1^ Department of Nephrology, Huai'an Second People's Hospital and The Affiliated Huai'an Hospital of Xuzhou Medical University, Huai'an, Jiangsu 223002, China; ^2^ Department of Emergency, Huai'an First People's Hospital, Nanjing Medical University, Huai'an 223300, China; ^3^ Department of Cardiology, The Fifth People’s Hospital of Shanghai, Fudan University, Shanghai 200240, China; ^4^ Department of Thoracic Surgery, Huai'an Second People's Hospital and The Affiliated Huai'an Hospital of Xuzhou Medical University, Huai'an, Jiangsu 223002, China; ^5^ Jiangsu College of Nursing, Huai'an, Jiangsu 223001, China

**Keywords:** acute kidney injury, insulin-like growth factor binding protein2, resveratrol-loaded nanoparticles

## Abstract

This study aims to determine whether insulin-like growth factor binding protein2 (IGFBP2) is a useful biomarker for early diagnosis of acute kidney injury (AKI), evaluate the therapeutic effects of resveratrol-loaded nanoparticles (Res-NPs), and investigate the possible underlying mechanisms in a rat model of AKI induced by IRI. Forty male Sprague–Dawley rats were randomly divided into four groups (10 animals per group): sham, IRI control, resveratrol, and Res-NPs injection. Kidney injury and the effects of Resveratrol and Res-NPs were determined by histological examination, renal function, cell apoptosis profile, and gene expression. Changes in IGFBP2 were similar with the pattern of well-known renal biomarkers, namely, kidney injury molecule 1 and neutrophil gelatinase-associated lipocalin, in all groups. Compared with the IRI control and resveratrol groups, the Res-NPs groups displayed significantly reduced apoptotic rate, reactive oxygen species level, and malondialdehyde content, downregulated protein expression levels of *Caspase3* and Bax with increased antioxidant glutathione peroxidase level, and upregulated expression of *Bcl-2* protein. Thus, IGFBP2 may serve as a promising novel biomarker of AKI, and Res-NPs may prevent kidney injury from ischemia/reperfusion in a rat model.

## INTRODUCTION

Acute kidney injury (AKI), is defined as acute reduction in glomerular filtration rate occurring over hours to days, with a subsequent rise in serum creatinine concentrations. AKI is a common and serious complication among a large range of human diseases characterized with a rapid decline in kidney function. The incidence of AKI in hospitalized population employing a Kidney Disease: Improving Global Outcome-equivalent criteria for AKI diagnosis was from 16.9% to 31.0% in Western countries [1] and from 7.5% to 31.0% in Asian areas [2]. Importantly, as much as 67.2% of intensive care children and young patients were diagnosed with AKI [3], which represents a significant medical and socioeconomic burden worldwide. Additionally, with its increase in the older population, the prevalence of AKI will continue to rise. However, early diagnosis of AKI is often difficult and its therapy is also lacking.

Discovering possible molecular biomarkers with the early diagnostic value is essential for prevention and intervention of AKI because conventional diagnosis of AKI mainly depends on delayed markers of kidney damage, such as oliguria and the rise of serum creatinine level. Recent study found that several promising molecular biomarkers, such as kidney injury molecule 1 (KIM-1) and neutrophil gelatinase-associated lipocalin (NGAL), can best reflect kidney and tubular function during early stage of AKI [4]. Moreover, the novel potential biomarkers are desperately needed to improve AKI outcome. Insulin-like growth factor binding proteins (IGFBPs) are a superfamily member of homologous proteins that are responsible for mediating insulin-like growth factor (IGF) bioavailability and activity. In view of the critical role of IGFBPs, IGFBP7 was found as an independent candidate biomarker for early detection of AKI with great sensitivity and specificity [5]. Likewise, IGFBP2 has been shown to be involved in the pathogenesis of metabolic diseases and carcinoma [6]. Moreover, silencing IGFBP2 can dramatically attenuate toxicity-induced cell necrosis and apoptosis [7], suggesting that it is closely related with cell injury. Therefore, we hypothesize that IGFBP2 may be a new potential biomarker for early diagnosis of AKI with excessive reactive oxygen species (ROS) triggering apoptosis in renal tubular epithelium.

As AKI not only affects short-term kidney function but also increases the risk of end-stage renal disease, thus to adopt timely interventions, such as volume repletion and treatment of associated infections [8], is important. In addition to therapies mentioned above, traditional Chinese herbal medicines for instance Curcumin [9] and Resveratrol [10] have been used to treat AKI. Although resveratrol has been demonstrated to possess potent nephrotective effect, low water solubility limits its application [11]. Our previous study showed that encapsulation in nanoparticles dramatically enhanced the water solubility of resveratrol and exerted neuroprotective effects on hydrogen peroxide-induced oxidative stress *in vitro* [12]. Thus, we continue to use resveratrol-loaded nanoparticles (Res-NPs) to treat rat model of AKI induced by ischemia/reperfusion injury (IRI) *in vivo*.

The present study, therefore, aimed to determine whether IGFBP2 is a useful biomarker for early diagnosis of AKI, and evaluate the efficacy of Res-NPs against ischemia/reperfusion (IR)-induced AKI, as well as investigate the possible underlying mechanisms.

## RESULTS

### Improvement of renal function after Res-NPs injection

**Figure 1:**
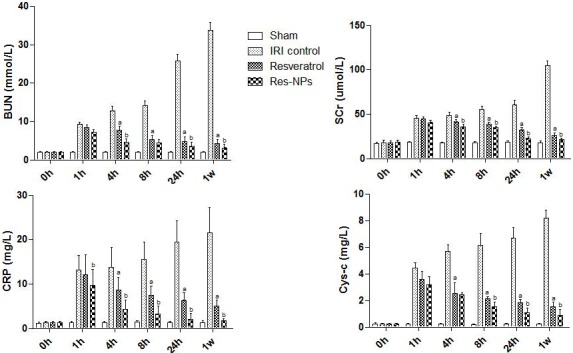
Renal function assay BUN, blood urea nitrogen; SCr, serum creatinine; CRP, c-reactive protein; Cys-C, cystatin Cprotease inhibitors; IRI control, ischemia/reperfusion injury control; Res-NPs, resveratrol-loaded nanoparticles; (**A**) the difference between IRI control and Resveratrol group is significant (*p* < 0.05); (**B**) there is a significant difference (Res-NPs VS Resveratrol) (*p* < 0.05).

To observe alterations in renal function, BUN, CRP, Cys-C, and SCr in serum were measured. Compared with animals in the sham group, Figure 1 indicates that after IRI, a dramatic reduction in the renal function of rat model was observed, suggesting that renal IRI model was successfully established. However, animals in the control group showed a significant continuous rise in serum levels of BUN, CRP, Cys-C, and SCr over time compared with the treated groups. Resveratrol and Res-NPs injection attenuated renal function impairment induced by ischemia. Furthermore, a significant difference in renal function between the Resveratrol and Res-NPs groups was observed (*p* < 0.05) (Figure 1A–1C and 1D).

### Histological changes in the kidney with IRI and effects of Res-NPs

**Figure 2:**
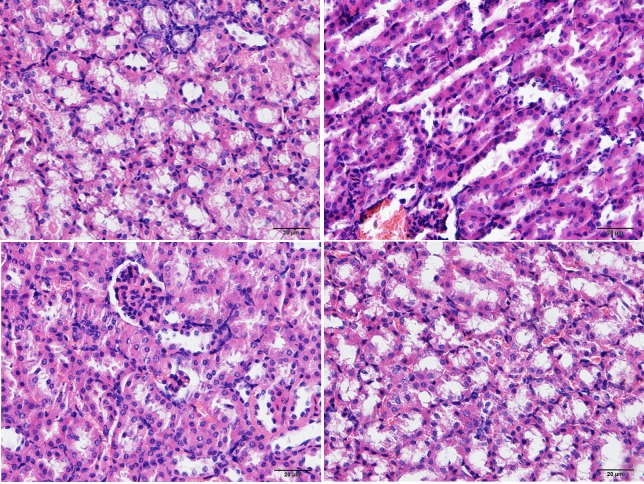
Haematoxilin/eosin staining Compared to Sham group (**A**), histological examination at 1 week revealed that animals in the IRI control group (**B**) subjected marked renal injury, such as cast formation, vacuolization, and tubular necrosis, and beneficial effects given by Resveratrol (**C**) and Res-NPs (**D**), (200×). Scale bars, 20 μm.

Histological examination at 1 week revealed that animals in the control group subjected marked renal injury, including cast formation, vacuolization, and tubular necrosis, compared with the sham group. Resveratrol and Res-NPs treatment contributed to a significant reduction of kidney injury a week after IR injury. However, the difference of the tubular injury score was significant between the Resveratrol and Res-NPs groups (Figure 2A–2C and 2D).

### Immunohistochemical staining for apoptotic index in the kidney

**Figure 3:**
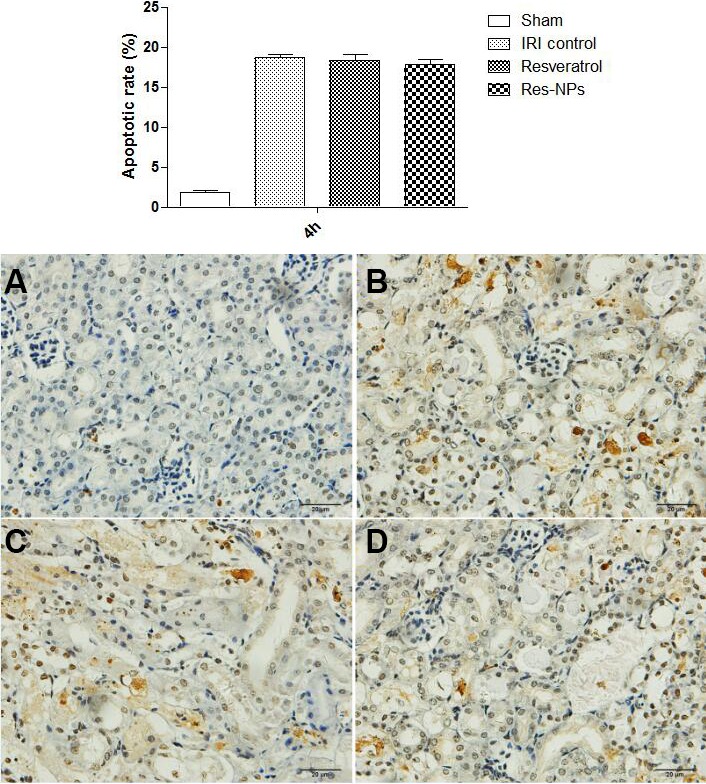

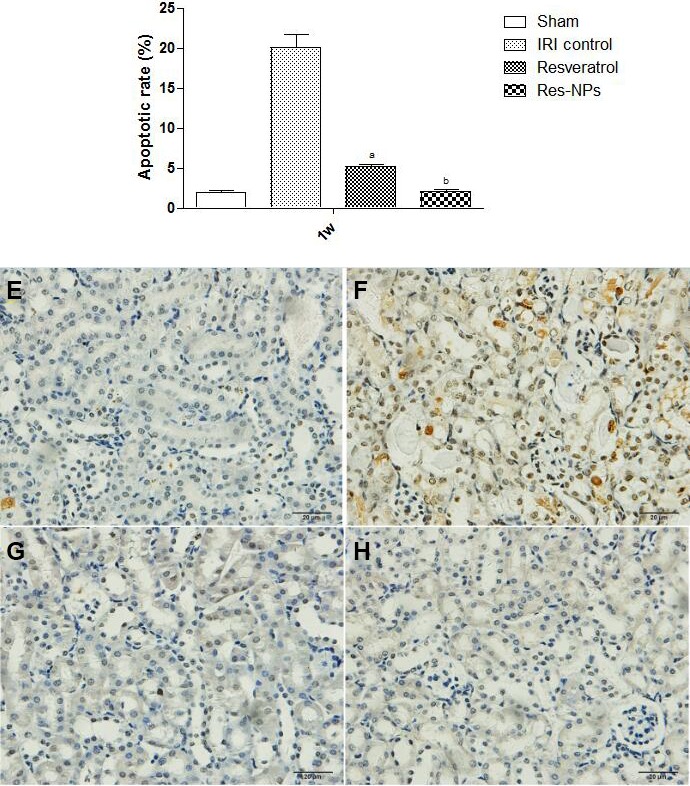
TUNEL assay The representatives of apoptosis rate at 4 h are showed in (**A**–**C**) and (**D**) (Figure 3A); (**E**–**H**) and h indicate the data at 1 week ((Figure 3B); (A) the difference between IRI control and Resveratrol group is significant (*p* < 0.05); (B) there is a significant difference (Res-NPs VS Resveratrol) (*p* < 0.05).

Cell apoptosis was evaluated by using TUNEL assay in the kidney sections. The number and proportion of TUNEL-positive cells were significantly increased in animal model from renal IR injury at 4 h (Figure 3A–3D), suggesting that severe apoptosis occurred. The results of TUNEL assay at one week showed that apoptosis was alleviated by both Resveratrol and Res-NPs compared with the control group, and significantly decreased apoptosis was observed in the Res-NPs group than in the Resveratrol group (Figure 3B–3H).

### Effects of Res-NPs on apoptosis of lymphocyte

**Figure 4:**
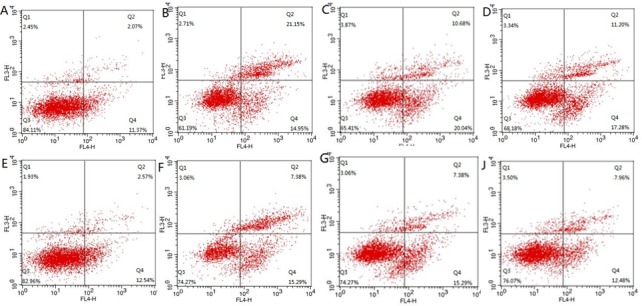
The change of apoptosis of lymphocyte detected flow cytometry (**A**–**C**) and (**D**) indicate representative of the results at 4 h after constructing model; (**E**–**G**) and (**H**) display apoptosis rate at 1 week; (A and E) Sham group, (B and F) IRI control, (C and G) Resveratrol, (D and H) Res-NPs.

To further determine the effects of Res-NPs injection, the levels of apoptosis in lymphocyte were assessed using flow cytometry. Data derived from flow cytometry profiles were qualitatively consistent with the results of replicate experiments. As displayed in Figure 4B and 4F, the control group showed a significantly increased number of apoptotic cells 4 h and one week after establishment of renal IRI model (36.1% and 46.45% in the IRI control group vs. 13.44% and 15.11% in the sham group). The number of apoptotic cells at 4 h and one week after constructing model gradually decreased in the Resveratrol and Res-NPs groups (Figure 4). The percentages of cells in the early apoptotic phase (4 h) were 36.1%, 30.72%, and 28.48% in the IRI control, Resveratrol, and Res-NPs groups, respectively. The corresponding percentages of cells in the late apoptotic phase (one week) were 46.45%, 22.67%, and20.44%. Data suggested that Res-NPs can prevent lymphocyte from apoptosis than Resveratrol.

### Effects of Res-NPs on GSH-Px and MDA

**Figure 5:**
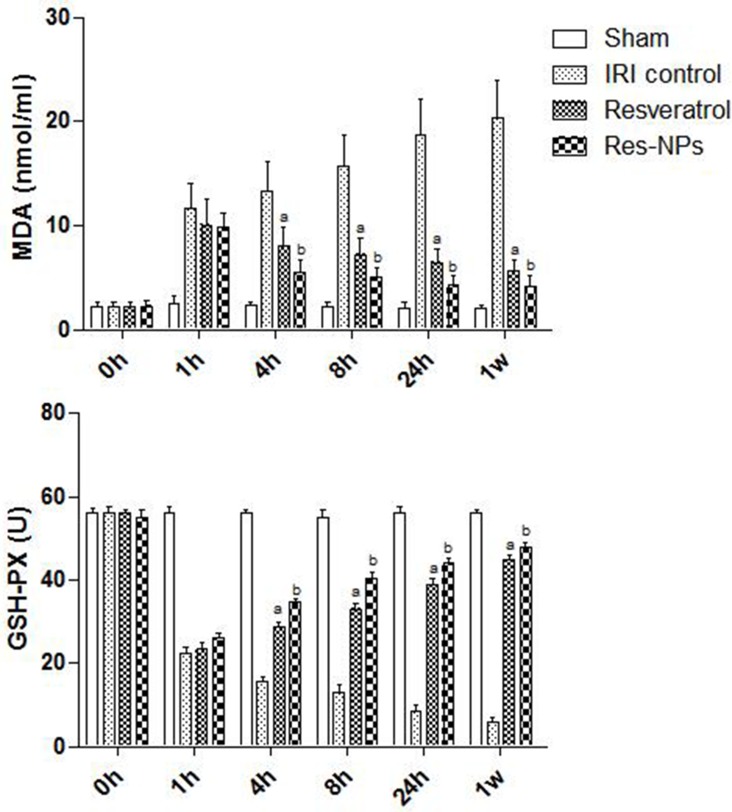
MDA and GSH-PX tests (**A**) the difference between IRI control and Resveratrol group is significant (*p* < 0.05); (**B**) there is a significant difference (Res-NPs VS Resveratrol) (*p* < 0.05).

GSH-Px and MDA levels were determined spectrophotometrically. As shown in Figure 5A, MDA levels of renal tissue were significantly increased in the groups treated with blocking the left kidney pedicle, whereas the respective GSH-Px levels markedly dropped. However, the elevation of tissue MDA levels was significantly suppressed when Resveratrol and Res-NPs administration followed IRI, and data suggested that Res-NPs can obviously reduce MDA production by inhibiting lipid peroxidation than Resveratrol. Additionally, treatment of Resveratrol and Res-NPs significantly elevated the reduced GSH-Px contents in the renal tissues compared with the control group, and such increase was significantly greater in the Res-NPs group than in the Resveratrol group (Figure 5B). These data above indicated that Res-NPs significantly reduced the MDA content and increased the GSH-Px contents (Figure 5).

**Figure 6:**
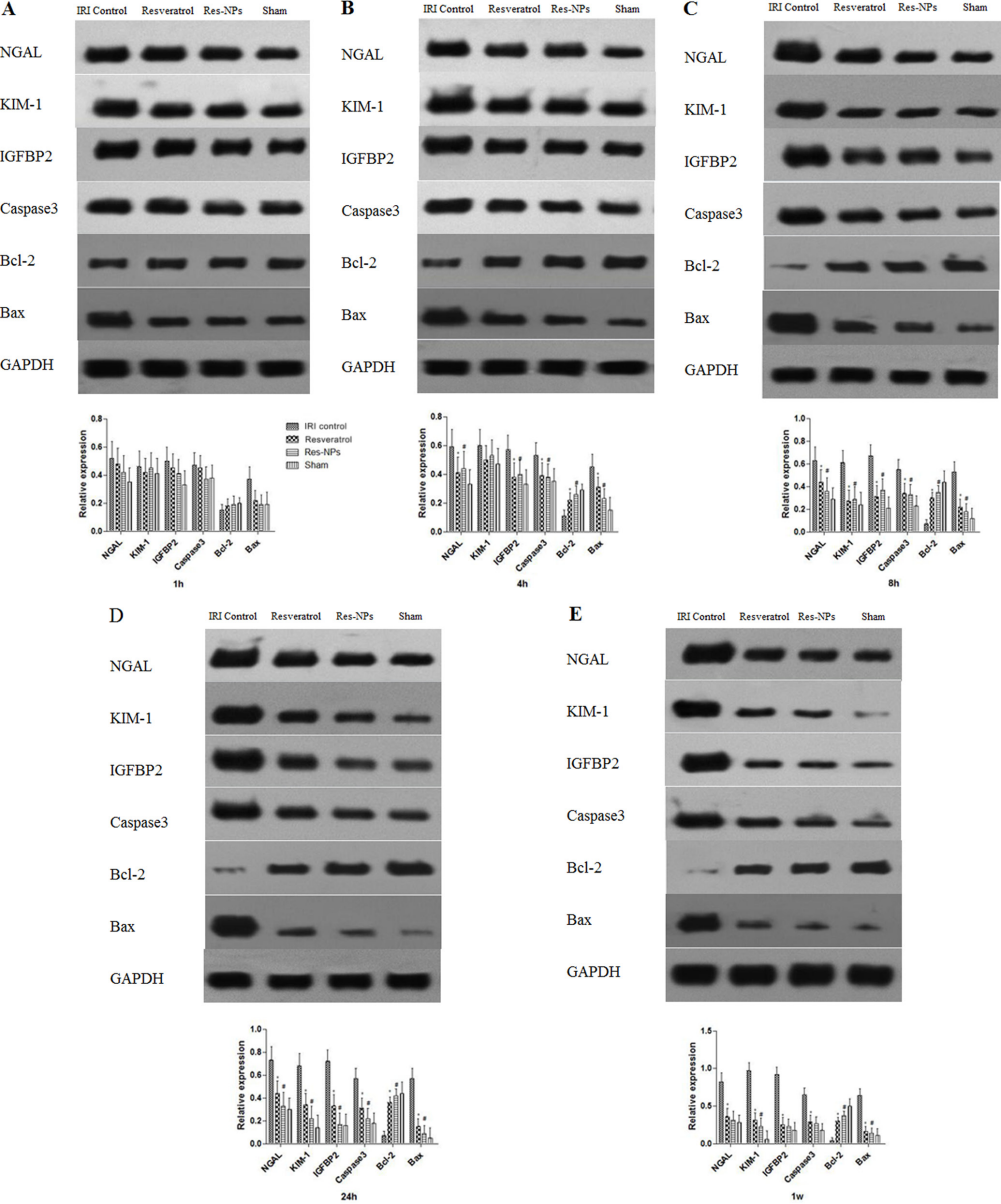
Changes in Expression levels of NGAL, KIM-1, IGFBP2, Caspase-3, Bcl-2, and Bax proteins in each group (**A–D**) and (**E**) represent 1, 4, 8, 24 h and 1w, respectively).

Alterations in the expression of *Igfbp2, Kim-1, Ngal, Bax, Caspase3*, and *Bcl-2* Expression levels of *Igfbp2, Kim-1, Ngal, Bax, Caspase3*, and *Bcl-2* proteins in each group were detected by Western blot assay (Figure 6A, 6B–6D, and 6E). The expression levels of *Igfbp2, Kim-1*, and *Ngal* were significantly (*p* < 0.05) higher in the renal IRI groups than that in the sham group at the initial period. However, the Resveratrol and Res-NPs groups exhibited significantly less expression over time than the control group. Importantly, the temporal trends of *Igfbp2, Kim-1*, and *Ngal* expression in all groups were similar, meaning that IGFBP2 can timely reflect renal injury profile.

Significantly increased levels of *Bax* and *Caspase3* protein expression were observed in the control group compared with the sham group. By contrast, expression of the antiapoptosis gene *Bcl-2* was significantly reduced by AKI. Our results also showed that administration of Res-NPs or Resveratrol downregulated the *Bax* and *Caspase3* protein expression but upregulated *Bcl-2* expression. The effects of Res-NPs on anti or proapoptosis gene expression were more prominent than Resveratrol (*p* < 0.05).

## DISCUSSION

In the present study, we detected *Igfbp2* expression in early stage of AKI development and assessed the effects of Res-NPs on AKI. Our study clearly demonstrated for the first time that IGFBP2 like KIM-1 and NGAL may be a reliable diagnostic and prognostic biomarker for AKI. Meanwhile, our data showed that Cur-NPs significantly improve renal function and inhibit cell apoptosis in IRI rat model by enhancing antioxidant activity, downregulating the expression of proapoptotic genes (*Caspase3* and *Bax*) and upregulating the expression of antiapoptotic gene (*Bcl-2*).

As the traditional markers of renal injury like SCr and BUN are insensitive especially at the initiation phase of AKI, new biomarkers with previous and accurate diagnostic value are therefore warranted. In this study, in addition to IGFBP2, KIM-1 and NGAL were also evaluated because they were often chosen as potential early indicator assessment of the ischemic AKI [13]. KIM-1 and NGAL can serve as biomarkers in the initiation, maintenance, and recovery phases of AKI using rodent model [14]. This finding is consistent with our results in which KIM-1 and NGAL expression levels were increased triggered by ischemia and declined with the recovery of renal injury with the treatment of Resveratrol and Res-NPs injection. Interestingly, changes in IGFBP2 were similar with the pattern of KIM-1 and NGAL in a time-dependent manner in all groups, suggesting that it may be a novel biomarker for early detection of AKI. Our study revealed for the first time the peculiar pattern of IGFBP2 in renal IRI rat model. IGFBP2 as a key member of IGF family is bound to complicate in these processes because of the pivotal role of the IGF system in the regulation of biological processes, such as cellular proliferation, differentiation, and apoptosis [15]. Previous studies demonstrated that hypoxia strongly upregulated the expression of *Igfbp2* gene [16, 17], whereas hypoxia is at the core of the AKI induced by IRI. Our data indicated that the level of IGFBP2 can better reflect timely the renal injury than traditional serum biomarkers (BUN, CRP, Cys-C, and SCr), indicating that IGFBP2 exhibits a good sensitivity for kidney damage. However, the exact mechanism on how *Igfbp2* expression was induced by AKI remains to be clarified in this study.

The results from histological examination, renal function, and TUNEL assay showed that renal tubular epithelial cell subjected severe injury, which accorded with other similar studies [18, 19]. Resveratrol has been shown to be a radical scavenging agent able to reduce free radicals [20]. In a rat model of sepsis-induced acute kidney injury, many studies have demonstrated that resveratrol supplement showed protective effects against tubular epithelium damage by restoring renal microcirculation and scavenging excessive reactive oxidant species [21, 22]. Similarly, administration of Resveratrol in our study significantly alleviated acute kidney injury compared with injection of saline. Notably, Res-NPs at 4, 8, and 24 h, as well as one week significantly improved renal function and cell survival than Resveratrol. The difference may be due to the following facts: Resveratrol has low bioavailability because of its poor aqueous solubility and dissolution properties elevated concentration of serum resveratrol resulting from encapsulation in nanoparticles; in addition, our previous study showed that Res-NPs characterized with the controlled release keep a high blood concentration of resveratrol in a sustained manner that is significant in maintaining the bioactivity [12]; furthermore, the antioxidative capacity of Res-NPs was evaluated than Resveratrol [12, 23].

Oxidative stress has been identified as a key role in the mechanism causing kidney failure. Studies have shown that ROS are involved in various cellular signaling pathways and transcription factors including mitogen-activated protein kinases, nuclear factor-κB, proapoptosis *Bax*, and *Caspase3*, as well as anti-apoptosis *Bcl-2*, which can activate cell survival and/or cell death processes [24]. Although ROS at low to the modest level is essential for cell physiological functions, the excess ROS is detrimental to cellular functions and survival [25]. Our study confirmed MDA level in renal tissue increased and GSH-Px production was inhibited during the process of IR, meaning that lipid peroxidation aggravation and antioxidative activity decline. Proapoptosis genes (*Bax* and *Caspase3*) were activated, whereas antiapoptosis gene (*Bcl-2*) was inactivated in rat model of AKI, which is in line with previous studies [26]. Administration of Resveratrol or Res-NPs significantly reduced rate of apoptotic cell and MDA level, suppressed proapoptosis genes while increasing GSH-Px content, and enhanced antiapoptosis gene expression. This finding suggests that Resveratrol or Res-NPs attenuate kidney injury through scavenging excess ROS and enforcing antioxidative ability. Our previous study showed the neuroprotective effect of Res-NPs by opposing oxidative stress [12]. Yin and colleague found that in transgenic *Caenorhabditis elegans*, resveratrol-loaded nanoparticles significantly alleviated oxidative stress induced by radiation or amyloid [23]. Although the protective effects of resveratrol are enhanced via delivery by nanoparticles, to our best knowledge, our study is the first to apply Res-NPs to treat AKI and compare its effect with that of resveratrol.

In summary, data indicated that IGFBP2 possessed high sensitivity and specificity for the early diagnosis of AKI as KIM-1 and NGAL. However, clinical research must be conducted to elucidate IGFBP2 as renal biomarker with diagnostic value in clinical practice. As expected, Resveratrol and Res-NPs exerted protective effects against oxidative stress in rats exposed to renal IRI *in vivo*. Furthermore, Res-NPs more effectively exhibited protective effects than the free Resveratrol. Thus, Res-NPs may be developed as a promising potential protective agent against AKI, and further studies on the effect of Res-NPs must be conducted.

## MATERIALS AND METHODS

### Drugs, reagents, and instruments

Resveratrol (C14H12O3; > 98% pure) was purchased from Shaanxi Sciphar Biotechnology Co., Ltd. 1,2-Distearoyl-sn-glycero-3-phosphoethanolamine and polyethylene glycol were purchased from Sigma Inc. Res-NPs were constructed as described in our previous study [12]. Phosphate-buffered saline (PBS), saline, apoptosis detection kit (Annexin VFITC/PI), protein extraction kit, malondialdehyde (MDA) kit, glutathione peroxidase (GSH-Px) kit, terminal deoxynucleotidyl transferase-mediated deoxyuridine-triphosphate nick end-labeling (TUNEL) apoptotic detection kit, and primary antibodies (BAX, caspase3, and BCL-2) were all purchased from Nanjing Key Gen Biotech. Co., Ltd. Blood urea nitrogen (BUN), C-reactive protein (CRP), cystatin C protease inhibitors (Cys-C), and serum creatinine (SCr) were all obtained from Weiteman, Co., Ltd., (Nanjing, China).

The following instruments were used: automatic biochemical analyzer (Hitachi 7100, Japan), microplate reader (ELx800, BioTek, USA), flow cytometer (FACSCalibur, Becton-Dickinson, USA), inverted fluorescence microscope (IX51, Olympus, Japan), Western electrophoresis apparatus (164-5051, Bio-Rad, USA), spectrophotometer (UV-2540, Shimadzu, Japan), and gel imager (Gel Doc XR, Bio-Rad, USA). Primary antibodies (IGFBP2, AAab136494; KIM-1, ab47634; NGAL, and ab63929) were purchased from Abcam (Cambridge, UK).

### Animals

All animal procedures were approved by the Animal Experiment Committee of Xuzhou Medical University (Xuzhou City, China). A total of 60 male Sprague–Dawley rats weighing 180–200 g at the start of treatment and 200–220 g when the experiments were performed were purchased from Beijing Vital River Laboratory Animal Technology Co., Ltd. (Beijing, China). Animals were housed five per cage in a standard room with constant temperature (22°C) and humidity on a 12 h light/dark cycle and with food and water ad libitum.

### Establishment of renal IRI rat model and study design

Animals were anesthetized with 10% chloral hydrate (3 mL/kg body weight) by intraperitoneal administration. Establishment of renal IRI rat model was performed as described previously [27]. Briefly, the right kidney was removed by a midline abdominal incision, whereas the left kidney pedicle was separated and blocked using a nontraumatic vascular clamp for 45 min, followed by removal of the clamp to initiate the left kidney reperfusion. Establishment of renal IRI was considered successful when the kidney was observed turning from pale to red.

Animals were divided randomly into four groups: sham group (sham operated, *n* = 10); IRI control group (injection with saline, *n* = 10); Resveratrol group (injection with Resveratrol, *n* = 10); and Res-NPs group (injection with Res-NPs, *n* = 10). Drugs or saline was administrated by a single tail vein injection (40 μg/g) shortly after establishment of AKI. Samples (blood and renal tissue via needle aspiration) in each group were collected and measured at 0 h, 1 h, 4 h, 8 h, 24 h and 1w, respectively.

### Renal function

Blood samples were collected for measurement of BUN, CRP, Cys-C, and SCr values in all four groups of rats (*n* = 10 for each group). Quantification of BUN, CRP, Cys-C, and SCr concentrations was carried out by using standard laboratory equipment in our hospital.

### Histological examination and TUNEL assay

After slides of paraffin-embedded renal tissue section (5 μm thick) were obtained, sections were stained with hematoxylin and eosin and evaluated by light microscopy. TUNEL assay was conducted to quantify DNA fragmentation according to the manufacturer’s instructions. Briefly, the slides were deparaffinized and rehydrated and covered with 30% H_2_O_2_ for 15 min to annul the endogenous peroxidases, followed by incubation with complete labeling reaction buffer and antibody solution, each for 1 h and 30 min. Finally, diaminobenzidine (DAB) solution was added to initiate colorimetric reaction. A dark brown DAB signal indicates positive staining (apoptotic cells). At least 1000 cells were counted in each of eight separate low-power fields for each sample, and the percentage of TUNEL-positive cells was calculated.

### Lymphocyte apoptosis detection through flow cytometry

Lymphocyte extraction and purification from blood sample was conducted based on the standard protocol [28]. Subsequently, the lymphocyte suspensions at a concentration of 1 × 10^6^ cell/mL were prepared. A binding buffer (500 μL) was then added to suspend the cells. Prior to 5 μL of PI was added and mixed thoroughly into the mixture; the cell suspension was mixed thoroughly with 5 μL of Annexin V-FITC. The system was allowed to react in the dark for 5–15 min at 25°C. The apoptotic conditions were subsequently detected via flow cytometry.

### Determination of GSH-Px and MDA contents

For the evaluation of oxidant–antioxidant system, the renal tissue was washed thrice with PBS and then its homogenate was prepared. The GSH-Px and MDA contents were measured using the assay kit and its optical density determined through spectrophotometry according to the instruction manual.

### Detection of protein expression through Western blot assay

Western blot analysis was performed to detect the expression levels of *Igfbp2*, *Kim-1IM-1*, *Ngal*, *Bax Caspase3*, and *Bcl-2* in renal tissues from all groups. Proteins were extracted from frozen tissues. After determination of protein concentration with bicinchoninic acid method, 100 mg of proteins in each sample was loaded into 12% sodium dodecyl sulfate-polyacrylamide gel electrophoresis electrophoretic separation. The protein was electrotransfered into a polyvinylidene fluoride membrane, blocked by using a blocking buffer containing 20 mM Tris–HCl (pH 7.4), 150 mM NaCl, and 0.1% (v/v) Tween 20 plus 5% fatfree milk (w/v) for 1 h, followed by blocking with a primary antibody (1:500) at 4°C overnight, and then incubated with secondary antibody (horseradish peroxidase-labeled goat anti-rabbit or goat anti-rat antibody 1:1,000) for 2 h. The results were obtained using chemiluminescence method, and photographs were captured using a gel image analysis system.

### Statistical analyses

All data were expressed as the mean ± SD of four independent experiments. Multiple groups were compared by using one-way analysis of variance followed by Tukey’s honest significant difference test for post hoc comparisons. All statistical tests were two-sided, and *P* < 0.05 was considered statistically significant. The data were analyzed by using SPSS 17.0.
